# Validation of the Gaming Skills Questionnaire in Adolescence: Effects of Gaming Skills on Cognitive and Affective Functioning

**DOI:** 10.3390/ejihpe14030048

**Published:** 2024-03-19

**Authors:** Triantafyllia Zioga, Chrysanthi Nega, Petros Roussos, Panagiotis Kourtesis

**Affiliations:** 1 Department of Psychology, University of Central Lancashire, Lancashire PR1 2HE, UK; t.zioga@student.icps.edu.gr; 2 Department of Psychology, College for Humanistic Sciences ICPS, 12131 Athens, Greece; 3 Department of Psychology, The American College of Greece, 15342 Athens, Greece; cnega@acg.edu; 4 Department of Psychology, National and Kapodistrian University of Athens, 15772 Athens, Greece; roussosp@psych.uoa.gr; 5 Department of Psychology, The University of Edinburgh, Edinburgh EH8 9Y, UK

**Keywords:** videogames, adolescence, cognition, memory, executive functions, attention, language, verbal fluency, emotion recognition, empathy

## Abstract

Given the widespread popularity of videogames, research attempted to assess their effects on cognitive and affective abilities, especially in children and adolescents. Despite numerous correlational studies, robust evidence on the causal relationship between videogames and cognition remains scarce, hindered by the absence of a comprehensive assessment tool for gaming skills across various genres. In a sample of 347 adolescents, this study aimed to develop and validate the Gaming Skill Questionnaire (GSQ) and assess the impact of gaming skills in six different genres (sport, first-person shooters, role-playing games, action-adventure, strategy, and puzzle games) on cognitive and affective abilities of adolescents. The GSQ exhibited strong reliability and validity, highlighting its potential as a valuable tool. Gaming skills positively affected executive function, memory, overall cognition, cognitive flexibility, and emotion recognition, except for empathy. Various game genres had different effects on cognitive and affective abilities, with verbal fluency influenced mainly by sports, executive functions by action, strategy, and puzzle, and emotion recognition positively impacted by action and puzzle but negatively by sports and strategy games. Both age and gaming skills influenced cognitive flexibility, with gaming having a greater effect. These intriguing genre-specific effects on cognitive and affective functioning postulate further research with GSQ’s contribution.

## 1. Introduction

Adolescence is generally described as the transitional period from the onset of puberty until adulthood [1]. Puberty typically starts at around 11 to 12 years of age, with a slightly earlier onset in girls than boys, and lasts until 18 to 21 years of age [2]. Adolescence is generally divided into three periods: early adolescence, which usually starts at 11 and ends at 13; middle adolescence, spanning from 14 to 17 years of age; and late adolescence, which includes the final years from 17 to 19 [3].

The distinction between the stages of adolescence is not strictly based on chronological age, but also on the achievement of different biological, psychological, and social milestones, as well as cognitive growth and accomplishments [2]. Specifically, regarding cognitive development, early adolescence marks the transitional period from the simple and concrete thinking of childhood to the complex and logical thinking of adulthood, which includes reasoning, abstract thought processes, consideration of others’ viewpoints, and an increased tendency for questioning [3]. Middle adolescence builds upon earlier achievements, while moral development starts by creating an ethical code, developing thoughts about the future, and gradually forming self-identity [3]. Finally, late adolescence represents the culmination of cognitive development, assuming its adult form. It is characterized by more robust decision-making processes, moving away from egocentric childhood and early adolescent thoughts, and enhanced impulse control.

The cognitive and affective development of adolescents can be influenced by many factors, with parental involvement being one of the most prominent. Regarding the use of technology, the family environment and appropriate parental regulation of use can have a significant positive or negative impact on children’s developing social skills, relationships, and overall cognitive skills [4,5,6]. Parental education and knowledge can also play an important role in adolescents’ cognitive skills, with higher levels of schooling correlating with increased language abilities [7]. Additionally, place of residence and, in particular, economic disparities, rather than rural or urban characteristics, can influence certain aspects of adolescents, such as empathy [8,9,10]. However, research in this area remains limited.

### 1.1. The Effects of Gaming Skills on Cognitive and Affective Functioning

Gaming has gained widespread popularity, with recent estimates indicating that approximately one third of the world’s population is gamers [11]. In countries like the U.S.A., the prevalence of gaming in children and adolescents is even higher, with most dedicating at least one hour to playing video games daily [12]. Additionally, more than 90% of adolescents are gamers, ranging from casual to intense playing [13]. Most gamers are often men, but in recent years, this has shifted toward a more even proportion of men to women who identify as gamers [14]. Furthermore, the gamer culture incorporates a gamer identity, which refers to a player’s practices and choices regarding gaming, like the games they choose, how they play them, and their preferred gaming system [14].

Videogames, from the moment they started to spread, have intrigued people and scientists alike. Many have raised their concerns regarding the potential adverse effects of digital technologies on the developing brains of adolescents, who are typically the age group that uses them the most [15,16,17]. Most studies initially focused on the adverse outcomes of videogames, but recently, this is starting to shift as more and more research highlights their potential benefits [12]. Some studies showed a connection between videogame play and decreased academic performance [18,19], while other studies thoroughly documented associations between violent videogames and aggressive behavior in children [20,21,22,23,24,25]. However, research suggests that videogames for young people can have both positive and negative effects, depending on the type and extent of use, as well as the presence or absence of protective factors like family support or diminished relations with peers [26,27,28].

Several studies support the idea that videogames can enhance adolescents’ cognitive and affective abilities. Attention is one of the most common abilities whose enhancement is associated with videogames [29,30,31,32,33,34], especially with action or shooter videogames [35,36,37,38,39,40]. This covers different attention metrics, including attention shift [41] and attention speed [35]. Specifically, it was found that playing puzzle video games correlates with improved sustained attention, whereas playing action video games correlates with improved attention speed [42]. However, some studies propose that videogames may have a negative effect on attention [43,44]. Cognitive flexibility, another cognitive skill, is found to be enhanced by real-time strategy games, followed by first-person shooters (FPS) and internet games in general [45,46,47,48].

Visuospatial ability is another cognitive domain commonly reported to be improved by videogame play [32,42], especially in types of games like action, FPS, and puzzle games [36,49]. Additionally, research has indicated a “dose-related” enhancement of visuospatial abilities in relation to time played [50]. Memory is also an important cognitive function, and the results from videogame studies range from indecisive to positive [51,52,53]. Action and FPS games are again specifically mentioned by specific studies, as they seem to act in favor of improving memory [52,54].

Executive functions, a critical category of cognitive skills, have also been targeted for improvement through videogame play [51,55,56,57,58,59]. With regard to specific games, strategy, puzzle, and exercise games have been identified as improving executive functions [55,57,59]. One study found that commercially available entertainment-focused videogames are not enough to boost executive function skills, but purpose-designed games may be appropriate to facilitate an enhancement of them [60,61,62]. Generally, while playing videogames appears to improve cognition, there is not a consensus that they can improve cognitive performance [63,64]. In the domain of language, studies have either found no significant association with videogame play [65] or found that videogames caused a downgrade of language skills in adolescents [66].

Emotion-related skills are also investigated, with videogames appearing to contribute to their improvement [67,68,69,70,71,72]. However, excessive videogame play, especially in in massive multiplayer online role-playing games (MMORPGs) and FPS [73], may be related to problematic emotion regulation and difficulties in expressing emotions [70,74,75]. While some studies have not found a clear connection between videogame play and emotion [76], there are suggestions of a reduction in empathy [77,78,79], although prosocial videogames may counteract this effect and potentially help at-risk teens [80,81,82]. Playing cooperative games, role-playing games, and games made to enhance empathy have been found to have the potential to improve empathy further [82,83,84,85]. This is a significant finding as empathy is closely linked to attachment and can protect against the aggressive behavior brought on by violent video games [86,87,88].

However, the major body of the literature discussed above is based on observed correlations between cognitive and affective abilities and videogame play. More robust statistical tools like the analysis of variance and regression should be applied to uncover more valid findings that could be used as a basis for generalization or future research. Furthermore, differences in videogame genres may lead to differential effects on players’ cognitive abilities [89,90,91,92,93]. Some research indicates that five factors in each videogame determine how it will affect its players, with the factors being the content, the context, the structure, the mechanics, and the amount of play [83,84,85,86,94]. Other researchers have also added that the game environment, whether two- or three-dimensional, and engagement factor play a role in determining the effect on cognition [87,88]. Despite differences in genres, some studies suggest that FPSs and RPGs have similar benefits to cognitive skills [38]. However, most research has focused on only differentiating players from non-players or experts from amateurs using non-standardized questions. As a result, the range of expertise or the distinctions between people who play different gaming genres has not examined thoroughly. In this direction, the duration and frequency of videogame play are important, with a few articles indicating the existence of a frequency threshold of videogame play, after which there are no further improvements in cognitive and affective abilities [34,95,96]. In contrast, long-term engagement with videogames, with a reasonable amount of time per week, indicates significant cognitive improvements [97,98]. Thus, the frequency of videogame play should be investigated to pinpoint further its relationship with players’ cognitive and affective abilities.

### 1.2. The Gaming Skill Questionnaire (GSQ)

As gaming continues to grow in popularity, many studies have assessed how gaming interacts with a wide array of functions in people of all ages. However, there seems to be a lack of standardized tools that a researcher could use to quantitively assess a person’s gaming skills, especially if one needs comparable results across different studies. Thus, the Gaming Skill Questionnaire (GSQ) was developed to assess a person’s level of expertise quickly and efficiently in gaming generally, as well as in different genres of gaming. The questionnaire comprises questions regarding the frequency of gaming activity and the self-perceived skill level in each genre. In assessing gaming experience, both skill level (how proficiently a player can navigate or control a game) and play frequency (how often a player engages with a game) are crucial metrics [99,100]. The initial inquiry focused on gauging the participants’ expertise (for instance, rating 5 for highly skilled) in using gaming systems, including VR setups and computers for gaming purposes. The subsequent query aimed to determine the regularity of their gaming activities (for example, rating 4 for weekly gaming sessions). This dual-measure approach, evaluating both proficiency and engagement, is recognized as an effective strategy for quantifying an individual’s experience with gaming technologies [99,100,101,102,103].

Furthermore, both frequency and ability of playing have been stated to be important in modulating how videogame play affects the different abilities of the players [16,34]. The GSQ includes six genres that are both widely popular and frequently investigated in studies [34,104,105]. These are: (a) sports games, encompassing racing games that feature a variety of vehicles, fighting games, and games featuring sports; (b) first-person shooter games, including games in which the player experiences the game environment through the perspective of the in-game character; frequently involved in shooting, while also incorporating Battle Royale type of games, which have gained considerable popularity in recent years. FPS games have generally been one of the most commonly researched gaming genres, as their inherently violent nature has intrigued researchers and the public since their introduction [6,106]; (c) role-playing games (RPGs), a broad category, incorporating both single-player and multiplayer games in which players assume roles, develop characters, and make decisions along the way. A popular subgenre of RPGs is massive multiplayer online RPG (MMORPG), characterized by the simultaneous existence of many players on a server. These games have been the research targets for their focus on teamwork and their commonly addictive nature [73]; (d) action-adventure games, another broad genre that involves games featuring exploration, a storyline, some combat, and/or some platforming aspects; (e) strategy games, varied in form, range from city builders to military games, and can be either real-time or turn-based. Finally, (f) puzzle-solving games include mystery games and traditional puzzle games. Strategy and puzzle games have been around in non-videogame form for centuries. However, due to their nature, they continue to intrigue researchers on how they affect the cognition of their players and how they compare to non-players in different abilities.

### 1.3. Aims of This Study

This study strived to achieve two goals. The first was the development and validation of a comprehensive tool, the Gaming Skill Questionnaire, designed to assess the gaming skills of a person by incorporating questions regarding gaming frequency and self-perceived expertise in games of different genres. The study rigorously examined the psychometric properties of GSQ, specifically focusing on its reliability and validity, to establish its robustness and reliability for future studies. This was achieved by performing Confirmatory Factor Analysis to assess construct validity (convergent and divergent), and Cronbach’s alpha to examine internal consistency. Secondly, in the process of test validation, the present study undertook a thorough exploration of the effects of gaming skills on cognitive affective abilities in adolescents: language skills, verbal fluency, executive function, memory, attentional speed, attentional shifting, and mental flexibility, emotion recognition and empathy. Finally, the study set out to investigate the relationship between gaming skills and the selected demographic variables of age, gender, residence location, parent age, parent education, and family income.

## 2. Materials and Methods

### 2.1. Participants

An *a priori* power analysis was conducted to determine the required sample size for the confirmatory factor analysis, which is the most demanding analysis required for the study’s aims. An online calculator specific to confirmatory analysis was used [107]. The calculator considers the fit indices for structural equation modelling (see also Confirmatory Factor Analysis subsection below) which have been suggested as central to examining structural validity of theoretical models and questionnaires [108,109]. The following minimum characteristics were used to determine the minimum sample size: (1) CFI = 0.9; (2) number of Items = 12; (3) number of Factors = 6; average factor loading = 0.65; average factor correlation = 0.05; significance (α) = 0.05; and power (1 − β) = 80%. Using the aforementioned characteristics, the *a priori* power analysis for Confirmatory Factor Analysis indicated a minimum sample size of 278 participants. We strived to recruit substantially more participants than this lower threshold to further ensure the best possible fit indices for assessing the validation of GSQ.

The study sample comprised 347 adolescents, evenly distributed across genders, with 167 boys and 180 girls. The participants, aged between 12 and 16 years, were students attending schools in the Macedonia (Thessaloniki and surrounding areas) and Attika (Athens and surrounding areas) regions of Greece. The educational level of the participants varied, encompassing those who had completed between 6 and 10 years of education. In Greece, that includes students from the 1st grade of gymnasium to 1st grade of lyceum. Geographically, the residences of these adolescents spanned a diverse range of areas, from smaller villages to larger urban areas with populations exceeding 100,000. This included adolescents living in small towns, larger towns, and large cities.

The sampling process was conducted using a random snowball sampling technique, and data collection began in May 2023 and was completed by mid-November 2023. Participation in this study was voluntary. A key inclusion criterion was that none of the participants had any diagnosed neurodevelopmental or attention disorders or any significant learning or language difficulties or disabilities. These criteria were set to ensure the collected data accurately reflected the cognitive and emotional profiles of typically developing adolescents in these Greek regions.

### 2.2. Materials

Participants’ socio-demographic information was collected through a custom questionnaire that encompassed queries about the adolescent’s sex, the age of both the adolescent and the parent, the education level of the parent, family income, and residence location (e.g., village, town, and city).

#### 2.2.1. Gaming Skills Questionnaire

The questionnaire was structured to evaluate the participants’ gaming habits and expertise across various video game genres. The questionnaire is structured into six sections, each focusing on a different game genre: sports, first-person shooter, role-playing, action-adventure, strategy, and puzzle solving. Each section contains two questions: one about the frequency of playing games from that genre and the other about the player’s self-assessed skill level in that genre. The frequency question offers options ranging from “Never” to “Every day”, and the skill level question uses a scale from 1 (No skill) to 6 (Expert).

In the Sports Games (SpG) section, participants reflected on their engagement with team sports video games, such as FIFA and PES, racing games like Need for Speed, and fighting games, including Tekken. They assess how frequently they play these games, from ‘Less than once a month’ to ‘Every day’, and rate their ability from ‘No experience’ to ‘Expert’.

The questionnaire then shifts to first-person shooting Games (FPSG), asking participants to reflect on their gaming frequency and skill level in games like Call of Duty and battle royale games like Fortnite. The same scales employed in SPG were used to evaluate gameplay frequency and proficiency level.

Participants again rated their play frequency and skill level for role-playing games (RPG), including titles like Skyrim, and MMORPGs, like World of Warcraft. This pattern is repeated across the remaining categories: action-adventure games (AAG), like Pokémon and Assassin’s Creed; strategy games (StG), including Age of Empires and StarCraft; and puzzle solving games (PSG), such as Portal and Inside. Participants were asked to evaluate how often they played these games and their self-perceived skill level in each category.

The GSQ produces six skill scores per game genre (i.e., subscores) and a total gaming skill score. The skill score for each game genre is calculated by adding the frequency and ability ratings for each game genre. The total gaming skill (TGS) score is then calculated as the sum of these individual scores, providing a comprehensive overview of the participants’ overall gaming engagement and expertise across different genres. This methodical approach allows for a detailed analysis of gaming patterns and proficiency levels, offering valuable insights into the gaming behaviors of the participants. The GSQ (English version) can be accessed here: http://dx.doi.org/10.13140/RG.2.2.27257.24160 accessed on 10 January 2024 (see also Supplementary Material).

#### 2.2.2. Edinburgh Cognitive and Behavioural ALS Screen (ECAS)

The Edinburgh Cognitive and Behavioural ALS Screen (ECAS) is a comprehensive assessment tool designed to evaluate a range of cognitive abilities [110]. This tool was selected for its ability to provide a total cognitive ability score and individual subscores across five distinct cognitive domains: language, verbal fluency, executive function, memory, and visuospatial ability. For this study, a Greek-adapted version developed and validated by Kourtesis et al. [111,112] was used. Specifically, the following domains are evaluated:

Language: The language component of the ECAS consists of three exercises with a total score of 28. These exercises entailed recognizing drawings depicting various items or animals, answering questions related to these drawings, and spelling a set of 12 words.

Verbal Fluency: The verbal fluency segment included two exercises, each with a total score of 24. Participants are asked to generate as many words as possible, starting with a specific Greek letter (Σ in one task and Π in the other) within 60 s, excluding proper nouns, place names, and numbers. The efficiency in this task is quantified by a Verbal Fluency index (VFi), calculated based on the number of words generated and the time taken to read them.

Executive Function: This section consisted of three exercises with a cumulative score of 48. It includes a reverse digit span task in which participants repeat number sequences in reverse order, an alternation task requiring switching between numbers and letters, and a sentence completion task in which participants complete sentences with irrelevant words. Additionally, there is a preference indication exercise that involves sets of pictures.

Memory: The memory component, scored out of 24, involved several exercises. Participants listened to a story and recalled as much information as possible immediately and after a delay. This delayed recall was designed to test memory retention.

Visuo-Spatial Ability: Scored out of 12, this section included exercises such as counting dots within rectangles, estimating the number of blocks in pseudo-three-dimensional drawings, and matching numbers in a rectangle to a corresponding dot’s position.

The total score of the ECAS was calculated by adding the subscores from these five cognitive domains, with a maximum possible score of 136. This comprehensive scoring system enables a profound understanding of the participants’ cognitive abilities across multiple dimensions.

#### 2.2.3. Trail Making Test (TMT)

The Trail Making Test (TMT) is a cognitive assessment tool used in this study to evaluate two specific aspects of cognitive function: attentional processing speed and cognitive flexibility [113,114]. This test was divided into two parts, each targeting different cognitive skills.

Part A—Attentional Processing Speed: In this section, participants are required to connect a sequence of numbers from 1 to 25 in order. The task involved using a pen to trace a line from one number to the next in ascending order. The primary objective of this part of the test was to assess the participant’s speed of processing information, particularly how quickly they can focus their attention and move from one task to another.

Part B—Cognitive Flexibility: This part of the TMT is more complex and is designed to evaluate cognitive flexibility. Participants were instructed to connect numbers and letters interchangeably (e.g., 1-A-2-B, etc.), starting from 1 and continuing up to 13. This task requires the participant to switch back and forth between two different sequences (numbers and letters), which challenges and measures their ability to mentally shift gears and adapt to changing rules and demands.

For both parts of the TMT, the time taken to complete the task from start to finish was recorded, which indicates the participant’s reaction time. Additionally, the number of errors made during the task was documented. These two metrics—time to completion and error count—provide valuable insights into the participants’ cognitive processing speed and flexibility.

This study focused on the basic metrics of time and accuracy, standard measures for assessing the cognitive functions targeted by the TMT. By including both parts of the TMT, A and B, the study aims to capture a more comprehensive picture of the participants’ cognitive abilities related to attention and flexibility.

#### 2.2.4. Reading the Mind in the Eyes Test (RMET)

The Reading the Mind in the Eyes Test (RMET) is a psychological tool designed to assess the ability of individuals to understand and interpret emotions and mental states through facial cues, explicitly focusing on the eye region [115,116]. This test has been widely used in various psychological and cognitive studies to evaluate the theory of mind and emotion recognition skills. In its standard format, the RMET includes 28 photographs depicting the eye region of different individuals. In addition to these photographs, an additional image is typically used for training purposes to help participants understand the test format. These images capture a variety of subtle and complex emotional states, conveyed solely through the eyes.

Participants undertaking the RMET were presented with these photographs one at a time. Each image contained four descriptive sentences or phrases. Their task was to select the sentence they believed most accurately described the emotion or mental state portrayed by the eyes in the photograph. This selection process required the participant to interpret subtle emotional cues and make judgements based on limited facial information. Scoring in the RMET is straightforward: each correct identification of the emotion or mental state associated with a photograph is awarded one point. Therefore, the maximum possible score a participant can achieve is 28, corresponding to the number of test images.

The RMET is recognized for its utility in studying social cognition, particularly in contexts where understanding subtle emotional cues is crucial. It has been employed in research related to autism, schizophrenia, and other conditions in which social cognitive impairments are a feature. The test is also valuable in general psychological assessments to measure an individual’s empathy level and ability to read emotional states from minimal cues. The RMET’s focus on the eye region is based on the premise that the eyes are a particularly expressive feature and can provide significant insights into a person’s emotional state, even without other facial or bodily cues.

#### 2.2.5. The Adolescent Empathy Spectrum Quotient (EQ)

The Adolescent Empathy Spectrum Quotient (EQ) is a comprehensive psychological assessment tool designed to evaluate various aspects of behavior and emotional functioning in young people [117,118]. This scale is tailored to capture the complex and often fluctuating behavioral patterns of the adolescent developmental stage. EQ encompasses a wide range of behavioral dimensions, including social skills, emotional regulation, impulsivity, and adaptability. Its design reflects an understanding of the unique challenges and changes that occur during adolescence, a period marked by significant psychological, emotional, and social development.

Administered in a questionnaire format, EQ invites adolescents to respond to a series of statements or questions about their typical behaviors, feelings, and attitudes in various situations. The scale is structured to provide insights into both positive aspects of adolescent behavior, such as resilience and empathy, as well as challenges like anxiety, mood fluctuations, and difficulties in social interaction. By covering a broad spectrum of behavioral and emotional domains, EQ offers a nuanced view of an adolescent’s psychological profile, aiding in identifying areas where support or intervention may be beneficial.

Scoring on the EQ involves evaluating responses to gauge the frequency, intensity, or severity of certain behaviors and emotional states. This scoring can help identify patterns or trends in behavior, providing valuable information for parents, educators, and mental health professionals. The scale is often used in educational and clinical settings to support the development of targeted strategies for addressing behavioral concerns, promoting mental well-being, and fostering positive adolescent social and emotional development.

In this case, the EQ-40 was used, which includes 40 questions answered by an adolescent’s parent. EQ is a valid (Confirmatory Factor Index = 0.93) and reliable (Item Reliability = 0.99) tool [118,119]. They were to choose between totally agree, somewhat agree, somewhat disagree, and totally disagree in each of the 40 question-statements regarding their adolescent child. For each question, one reply was scored as 2 points, one as 1 point, with the rest netting no point. The final score was the sum of the scores in each of the 40 questions.

### 2.3. Procedure

Each participant was interviewed individually to collect the necessary data. The interviews were conducted in a private and quiet environment. Before the interviews began, a detailed briefing was provided to the adolescents and their parents. This briefing included information about the nature of the tests to be conducted, the types of data that would be collected, and the strict confidentiality maintained throughout the process. Informed consent was obtained from participants and their parents before data collection was initiated.

Initially, participants completed the adolescent-focused section of the demographic questions and the GSQ. GSQ assessed the participants’ frequency and proficiency in playing videogame genres and overall gaming skills. During questionnaire completion, emphasis was placed on ensuring that participants provided accurate and thoughtful responses.

Subsequently, three cognitive and emotional assessments were administered: ECAS, TMT, and the RMET. To control potential order effects and participant fatigue, a Latin square design was used to randomize the sequence of these three assessments.

During the administration of these tests, the participants’ parents were not present to prevent any possible influence on the participants’ responses. Upon completing the three assessments, parents were invited to participate in the study by completing the parental section of the demographic and EQ questionnaires.

The data collection process ended with an expression of gratitude to both the participants and their parents for their invaluable contribution and cooperation in the study.

### 2.4. Statistical Analyses

Statistical analyses for this study were performed using the R programming language (version 4.3.3) [120] within the RStudio environment (version AGPL v3) [121]. Essential R packages were utilized, including psych [122] for correlation, regression, ANOVA, and post hoc comparison analyses and ggplot2 [123] for generating visual plots. The first step in our analysis involved descriptive statistics, providing a comprehensive overview of the sample demographics and test scores. We employed paired samples *t*-tests to investigate differences in key variables at various intervals. Multiple linear regression analyses were conducted to identify predictors of specific cognitive and behavioral outcomes, while mixed linear regression models were used to explore determinants of performance across a range of tasks. Due to deviations from normal distribution in our dataset, we applied transformations using the bestNormalize [124] package in R. This approach was crucial to achieve normal distribution in the data, thus facilitating the use of parametric statistical methods. The transformed data were centered to z-scores, allowing a more uniform analysis process.

#### 2.4.1. Confirmatory Factor Analysis

Confirmatory Factor Analysis (CFA) was employed to evaluate the structural and construct validity of the GSQ. This statistical method is crucial for verifying whether the data fits a hypothesized measurement model, in this case, the structure of the GSQ. The CFA aimed to ascertain the associations between the observed variables (items in the questionnaire) and their underlying latent constructs, represented by different gaming skill domains such as sport games skill (SpGS), first-person shooting games skill (FPSGS), role-playing games skill (RPGS), action-adventure games skill (AGS), strategy games skill (StGS), and puzzle games skill (PGS).

The GSQ’s structural validity was complemented by evaluating its goodness of fit using various statistical indices in the CFA. These indices included the Chi-Square to degrees of freedom ratio (χ^2^/df), Comparative Fit Index (CFI), Tucker–Lewis Index (TLI), Standardized Root Mean Residual (SRMR), and Root-Mean-Square Error of Approximation (RMSEA). These measures provide a comprehensive assessment of how well the model fits the observed data, with thresholds set for each statistic to determine an acceptable level of fit. To assess the internal reliability and consistency of the GSQ, Cronbach’s alpha coefficients were calculated for each domain. This measure of internal consistency is critical in evaluating the degree to which items within a scale are inter-correlated, thus indicating the scale’s reliability. A Cronbach’s alpha value of 0.70 or higher is generally considered acceptable, indicating good internal consistency of the scale or subscale.

#### 2.4.2. Regression Analysis Process

The regression analysis was initiated by verifying data normality using the Shapiro–Wilk Normality test and confirming homoscedasticity with the Non-Constant Error Variance test. Multicollinearity was assessed by calculating the variance inflation factor for each predictor within the models. Linear regression analyses were employed to explore various predictors of cognitive functions and behaviors. The models were compared using analysis of variance, with evaluation criteria including the Akaike Information Criterion (AIC), Bayesian Information Criterion (BIC), the F statistic and its significance level, and the proportion of explained variance (R^2^).

For the Linear Multiple Regression analyses, variables such as demographic factors (age, residence location, parent age, parent education, family income), gaming skills (SpGS, FPSGS, RPGS, AGS, StGS, PGS, TGS), and cognitive test scores (ECAS–language, ECAS–verbal fluency, ECAS–executive functions, ECAS–memory, ECAS–visuospatial, ECAS–total score, TMT–A–reaction time, TMT–B–reaction time, RMET, empathy quotient) were considered. An incremental approach was adopted for the analytical process:

Single-Predictor Models: Initially, separate models were developed for each predictor. The most effective variable was identified based on the performance of these initial models.

Dyadic Predictor Models: Subsequently, models incorporating two predictors were constructed, consistently including the most effective variable from the single-predictor models. The performance of these dyadic models was then evaluated and compared to that of the single-predictor models to ascertain the most effective combination.

Incremental Model Development: This approach was characterized by its iterative nature, where each subsequent phase involved adding a predictor and comparing the performance of increasingly complex models. This process was continued until the inclusion of new variables no longer significantly improved the models’ performance. The optimal model was determined when a simpler model from an earlier phase demonstrated superior performance compared to a more complex model from a later phase. This ensured that the final selected model was robust and accurately reflected the most influential variables identified in the study.

#### 2.4.3. Two-Way ANOVA Analyses

Two-way ANOVA Analyses were conducted on a balanced subset of the sample across various groups, with a total of 238 participants comprising 116 boys and 122 girls. The considered sample was evenly distributed across early and middle adolescence stages, as well as low and high gaming skill levels, with respective counts of early-low (*n* = 60), middle-low (*n* = 60), early-high (*n* = 59), and middle-high (*n* = 59). The subset for the ANOVAs was necessary to examine the potential effects of age (early and middle adolescence) and gaming skill level (low and high), and their interaction effect on the measured variables. By creating a balanced design with equal numbers in each category and subgroup, the study aimed to ensure that any detected differences or patterns in the data could be attributed to these specific factors without disproportionate influence from any single group.

Two-way ANOVA analyses were utilized to assess cognitive and affective functioning during different stages of adolescence and across varying levels of gaming skill. These analyses considered the adolescence stage (i.e., early and middle) and gaming skill level (i.e., low and high) as independent variables, and scores on the ECAS, TMT, RMET, and EQ as dependent variables. The effects of the adolescence stage, gaming skill, and their interaction were examined for each cognitive domain evaluated, such as language, verbal fluency, memory, executive functions, and overall ECAS score. Similarly, the TMT-A and TMT-B tests were analyzed to assess visual attention speed and mental flexibility, respectively. Bonferroni Corrected Comparisons were also conducted to investigate differences between specific groups further.

This comprehensive approach for performing two-way ANOVA analyses provided a detailed understanding of the influence of both developmental stage and gaming skill level on various cognitive and affective functions among adolescents. The balanced nature of the sample across different groups added robustness to the analysis, ensuring a representative examination of these factors.

## 3. Results

The sample (*N* = 347) comprised 167 boys and 180 girls aged 12 to 16. The descriptive statistics of their demographics, which include age, their residence location (ordinal variable by the size), the age of their parents and their education, and the family income, can be found in Table 1. This table also notes the descriptive statistics of the GSQ, ECAS, TMT, RMET, and EQ. The ECAS–Visuospatial score reached a ceiling effect, with the mean being equal to the maximum possible score of 12, with 0 standard deviation. Thus, it was not included in the subsequent statistical analyses.

### 3.1. GSQ Internal Reliability, Goodness of Fit, and CFA

Measures of the internal reliability and goodness of fit for the GSQ can be found in Table 2. Specifically, Cronbach’s alpha, which indicates internal reliability, was found to be excellent in each of the six sections (SpGS, FPSGS, RPGS, AGS, StGS, PGS), with values ranging from 0.80 to 0.91. Furthermore, goodness of fit was calculated using five different measures which include Chi-Square to degrees of freedom ratio (χ^2^/df), Comparative Fit Index (CFI), Tucker–Lewis Index (TLI), Standardized Root Mean Residual (SRMR), and Root-Mean-Square Error of Approximation (RMSEA). All five of the measures indicated a good fit for the current model. Specifically, χ^2^/df achieved a 1.97 with the threshold being under 2.00, CFI achieved 0.96 with the threshold being over 0.90, TLI achieved 0.91 with the threshold being over 0.90, SRMR achieved 0.08 with the threshold being under or equal to 0.08, and RMSEA achieved 0.08 with the threshold being equal to or under 0.08. It must be noted that SRMR and RMSEA achieved 0.798 and 0.779, respectively, with both being actually under the threshold of 0.08.

**Figure 1:**
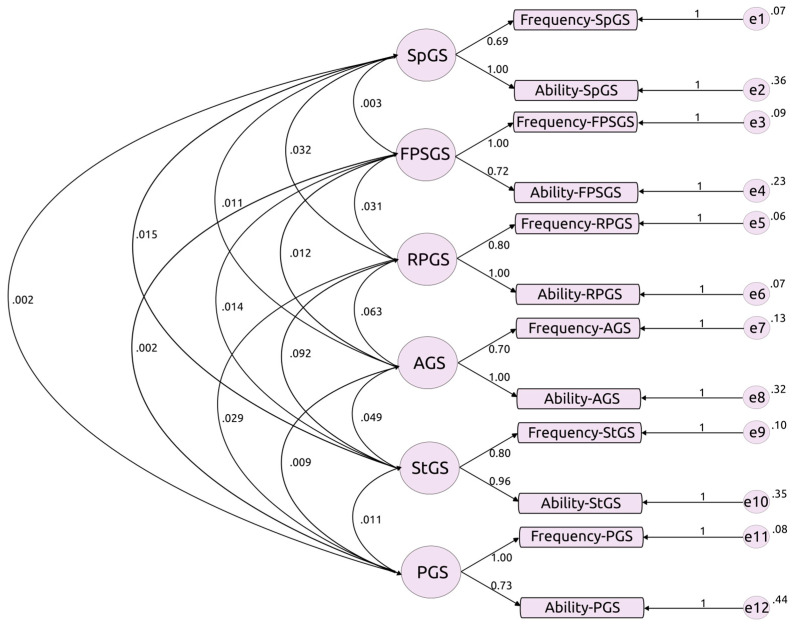
Confirmatory factor analysis path diagram: From left to right: the structural model illustrates the associations between GSQ factors and the items loadings to each factor. On the right there are the error items (e) for each item; SpGS = sport games skill; FPSGS = first-person shooting games skill; RPGS = role-playing games skill; AGS = action-adventure games skill; StGS = strategy games skill; PGS = puzzle games skill. Higher numbers indicate stronger associations.

The outcomes of the Confirmatory Factor Analysis (CFA) are visualized in Figure 1, where one can see the associations between the six different Skill sections, the association of each Skill to the questions of frequency and ability, and the error items for each item. All item loadings are high, with the lowest value being 0.69 and the highest being 1, indicating good to excellent convergent validity. On the other hand, the associations between the different sections of the GSQ are low, with the highest value being 0.092 and the lowest value being 0.002, indicating an excellent divergent validity.

### 3.2. Correlations

The Pearson’s r correlations are being reported in Table 3. Starting with demographic data, age appears to be strongly correlated with language, verbal fluency, ECAS total score, TMT-A and B, and weakly correlated with executive function, memory, and EQ. residence location was only weekly correlated with executive function, memory, total ECAS score, and EQ. Parent age was weakly correlated with language, verbal fluency, executive function, ECAS total score, and TMT A and B. Parent education was only weakly correlated with language, memory, and ECAS total score. Family income was only weakly correlated with TMT-B. All in all, the only strong correlations of the demographic data were age with language, verbal fluency, total cognition, attentional processing speed, and cognitive flexibility. It is important to note that in the context of measuring reaction times with TMT A and B, when there is a negative relationship between attentional processing speed (in the case of TMT-A) or cognitive flexibility (in the case of TMT-B) and the independent variables, this implies that as the independent variables increase, the reaction times decrease. This suggests an enhancement of attentional speed and cognitive flexibility, respectively.

Moving on to gaming skills, SpGS was weakly correlated with RMET and EQ. FPSGS was weakly correlated with executive function, memory, total ECAS score, and EQ. RPGGS, like FPSGS, was weakly correlated with executive function, memory, total ECAS score, and EQ. StGS was weakly correlated with the same as RPGS but also with TMT-B. AGS was moderately correlated with executive function and EQ and weakly correlated with memory, ECAS total, TMT-A and B, and RMET. PGS was moderately correlated with executive function and ECAS total score and weakly correlated with all the rest (language, verbal fluency, memory, TMT-A and B, RMET, and EQ). finally, total gaming skill was moderately correlated with executive function, memory, total ECAS score, and EQ, and weakly correlated with language, RMET, and TMT-A and B. Of all gaming skills, there were no strong correlations. However, there were moderate correlations between AGS and executive functions and empathy, between PGS and executive functions, and between TGS and executive functions, memory, total cognition and empathy.

### 3.3. Best Regression Models

Table 4 depicts the best regression models for each of the tests performed. The ECAS-language best model had an R^2^ of 0.50, indicating a moderate to strong relationship between the dependent variable of language and the predictors included, namely age and parent education. Of the two predictors, age had a beta coefficient of 0.68, meaning that an increase in age does lead to a substantial increase in language skills. At the same time, parental education had a beta coefficient of 0.13, indicating that an increase in parental education level would lead to a moderate increase in the adolescent’s language skills.

The ECAS-verbal fluency best regression model had an R^2^ value of 0.52, postulating a strong relationship between verbal fluency abilities of adolescents and age, residence location and SpGS. The strongest predictor was age, with a beta coefficient of 0.72, indicating that an increase in the age of adolescents leads to a large increase in their verbal fluency ability. Residence location has a small beta coefficient of 0.08, meaning that a change from a residence location with a smaller population to a larger one leads to a weak increase in verbal fluency. Lastly, SpGS had a beta coefficient of −0.11, meaning that if an adolescent has better gaming skills in sports games, it will lead to a medium-sized decrease in verbal fluency.

ECAS-executive function’s best regression model achieved an R^2^ of 0.21, indicating that executive function is moderately related to the predictors included, AGS, StGS, and PGS. AGS achieved a beta coefficient of 0.29, pointing out that an increase in AGS leads to a medium increase in executive functions. the same can be said for PGS and StGS, which have beta coefficients of 0.24 and 0.16, respectively.

The best regression model for ECAS-memory had an R^2^ of 0.20, which points out that the predictors, namely age, residence location, and TGS, have a low to moderate relationship with memory. Of the three predictors, TGS had a beta coefficient of 0.28, meaning that an increase in TGS leads to a moderate increase in memory. Residence Location had a beta coefficient of 0.23, meaning that living in a bigger population center is connected to a moderately better memory. Also, as age has a beta coefficient of 0.19, being an older adolescent lead to moderately better memory ability.

ECAS-Total Score’s best regression model has an R^2^ of 0.54, which indicates a strong relationship between it and the predictors. Said predictors include age, with a beta coefficient of 0.66; residence location, with a beta coefficient of 0.15; and TGS, with a beta coefficient of 0.21. These results indicate that an older age leads to a substantial increase in total cognition and that both living in a larger population center and having a higher TGS lead to a moderate increase in total cognition.

The best regression model of TMT-A achieved an R^2^ of 0.63, indicating a strong relationship between the scores of TMT-A and the sole predictor of the model, age. Age had a beta coefficient of −0.79, which indicates that an increase in age leads to a substantial improvement in attentional processing speed.

TMT-B’s best regression model has an R^2^ of 0.60, indicating a strong relationship between the predictors of the model, which include age, family income, and TGS, and TMT-B. The beta coefficient of age is −0.74, meaning that the older age of adolescents leads them to a larger increase in cognitive flexibility. Family income has a beta coefficient of −0.10, meaning that an increase in family income leads to a small to medium increase in cognitive flexibility. Lastly, for TMT-B, TGS has a beta coefficient of −0.11, indicating that an increase in TGS for adolescents leads to a moderate increase in cognitive flexibility.

The best predictive model of RMET included Residence Location, SpGS, AGS, StGS, and PGS as predictors, which appear to be weakly related to RMET, as the R^2^ was 0.10. Residence location had a beta coefficient of 0.11, pointing out the fact that living in a bigger population center leads to a moderate increase in emotion recognition ability. SpGS had a beta coefficient of −0.14, while StGS had a beta coefficient of −0.13, both indicating that an increase in SpGS or StGS leads to a moderate decrease in adolescent emotion recognition ability. On the other hand, AGS had a beta coefficient of 0.14, and PGS had a beta coefficient of 0.17, both pointing out that an increase in AGS or PGS leads to moderate increases in emotion recognition ability.

EQ’s best regression model had an R^2^ of 0.27, indicating a moderate relationship between empathy and the predictive factors, Residence Location and TGS. Residence Location had a beta coefficient of =0.21, showing that living in a place with a bigger population leads to moderately less empathy in adolescents. TGS had a beta coefficient of −0.45, meaning that an increase in an adolescent’s TGS leads to a substantial decrease in empathy.

### 3.4. ANOVA Analyses

The ANOVAs were conducted on a subset of the sample (N = 238), with 116 boys and 122 girls. These could be divided based on their age in early and middle adolescence with 119 participants in each group. Furthermore, they could be divided based on their gaming skills into low, with 120 participants, and high, with 118. Considering both age and gaming skill, this subsample can be divided into four groups: early low with 60 participants, middle-low with 60 participants, early high with 59 participants and middle-high with 59 participants. The subset was created to analyze the influence of age and gaming skill on cognition. By equally distributing participants across groups like early low, middle-low, early high, and middle-high, the study could isolate and understand the effects of these variables more accurately. The descriptive statistics for each of these four subgroups in cognitive and affective functioning are presented in Table 5.

**Figure 2:**
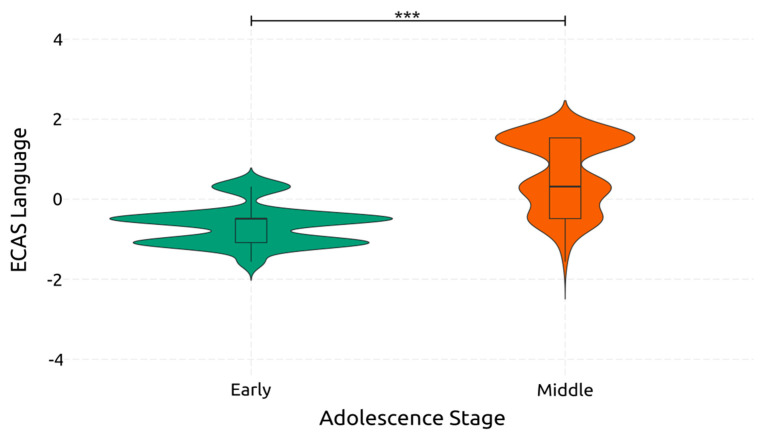
Comparison of early vs. middle-stage adolescence: language performance; *** *p* ≤ 0.001.

The ANOVA revealed a statistically significant and large effect of adolescence on language skills [*F*(1, 234) = 182.157, *p* < 0.001, *η*^2^*_p_* = 0.438], which is visualized in Figure 2. On the contrary, gaming skill had a non-significant effect [*F*(1, 234) = 0.441, *p* = 0.508, *η*^2^*_p_* = 0.002], and the interaction between the two had a non-significant small-sized effect [F(1, 234) = 3.187, *p* = 0.076, *η*^2^*_p_* = 0.013]. These results indicate that language significantly improves from early to middle adolescence. Conversely, gaming skill does not significantly impact the language ability of adolescents.

**Figure 3:**
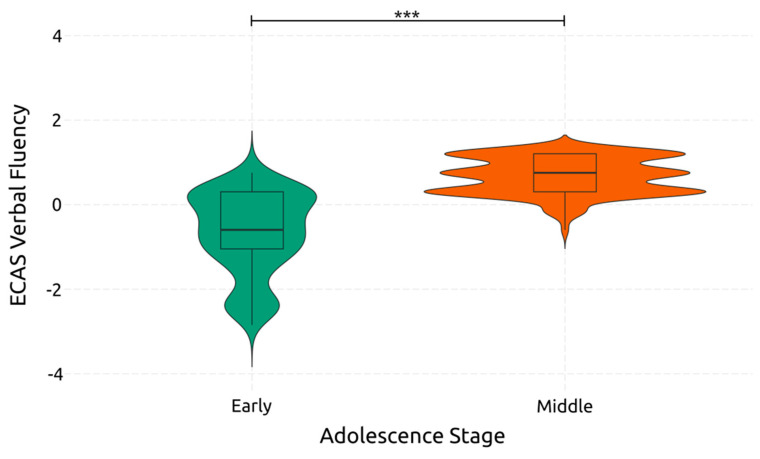
Comparison of early vs. middle-stage adolescence: verbal fluency; *** *p* ≤ 0.001.

Verbal fluency, like language, is significantly affected by adolescence [*F*(1, 234) = 200.044, *p* < 0.001, *η*^2^*_p_* = 0.461]. On the contrary, gaming skill did not significantly affect verbal fluency [F(1, 234) = 2.072, *p* = 0.151, *η*^2^*_p_* = 0.009]. The interaction between gaming skill and adolescence [F(1, 234) = 0.759, *p* = 0.385, *η*^2^*_p_* = 0.003] did not significantly affect verbal fluency either. Figure 3 visualizes the difference between the two adolescent groups regarding verbal fluency. These results indicate that verbal fluency improves when transitioning from early to middle adolescence. However, this is not the case for gaming, where high and low gaming skills do not cause significant differences in verbal fluency.

**Figure 4:**
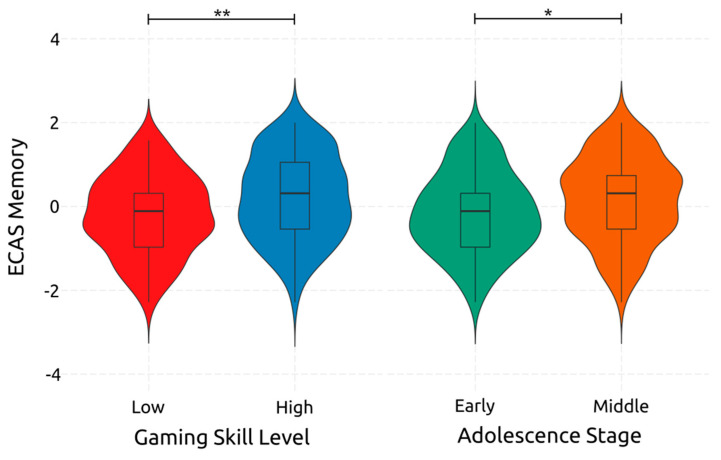
Comparison of low vs. gaming level and early vs. middle-stage adolescence: memory; * *p* ≤ 0.05, ** *p* ≤ 0.01.

Adolescence had a significant small-sized effect on memory [F(1, 234) = 6.320, *p* = 0.013, *η*^2^*_p_* = 0.026]. The same relationship can be observed with gaming skill on memory [F(1, 234) = 10.240, *p* = 0.002, *η*^2^*_p_* = 0.042]. However, the interaction between the two did not significantly affect memory [F(1, 234) = 1.220, *p* = 0.270, *η*^2^*_p_* = 0.005]. In Figure 4, one can see the visualized effects of both adolescence and gaming skill on memory. The results indicate that transitioning from early to middle adolescence leads to better memory skills and that having a high gaming skill leads to better memory.

**Figure 5:**
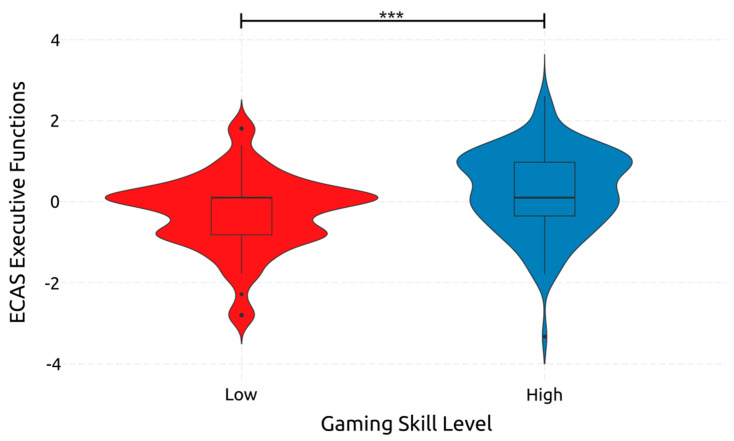
Comparison of low vs. gaming level: executive functions; *** *p* ≤ 0.001.

Moving on to executive function, gaming skill had a significant medium-sized effect [F(1, 234) = 19.640, *p* < 0.001, *η*^2^*_p_* = 0.077] which is depicted in Figure 5. Adolescence did not significantly affect executive functions [F(1, 234) = 1.827, *p* = 0.178, *η*^2^*_p_* = 0.008]. Neither did adolescent gaming skills [*F*(1, 234) = 0.254, *p* = 0.614, *η*^2^*_p_* = 0.001]. These results indicate that the age of adolescents does not significantly affect their executive function abilities, but higher gaming skills do lead to better executive function abilities.

**Figure 6:**
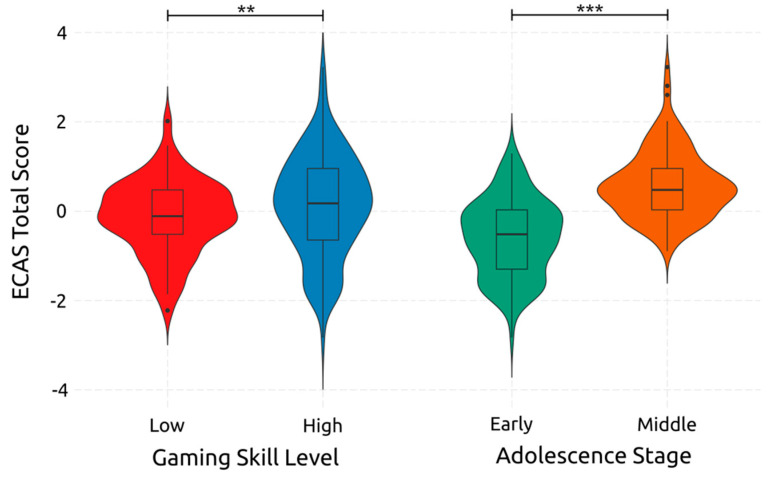
Comparison of low vs. gaming level and early vs. middle-stage adolescence: overall cognitive performance on ECAS; ** *p* ≤ 0.01, *** *p* ≤ 0.001.

ECAS Total Score was affected significantly by Adolescence, which had a large effect size [F(1, 234) = 130.510, *p* < 0.001, *η*^2^*_p_* = 0.358]. It was also significantly affected by Gaming skill, which had a small effect size [F(1, 234) = 7.000, *p* = 0.009, *η*^2^*_p_* = 0.029]. Both their effects are visualized in Figure 6. However, the interaction between adolescence and gaming skill had a non-significant small size effect [F(1, 234) = 2.43, *p* = 0.120, *η*^2^*_p_* = 0.010]. These results point out that when growing up from early to middle adolescence, total cognition is improved, and that improving one’s gaming skills leads to an increase in total cognition.

**Figure 7:**
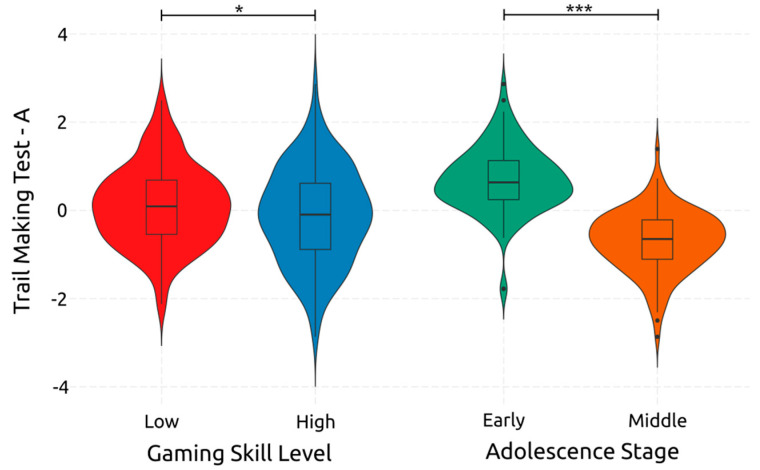
Comparison of low vs. gaming level and early vs. middle-stage adolescence: visual attention speed; * *p* ≤ 0.05, *** *p* ≤ 0.001.

TMT-A, and thus attentional processing speed, appears to be largely and significantly affected by adolescence [F(1, 234) = 230.310, *p* < 0.001, *η*^2^*_p_* = 0.496]. Gaming skill also has a significant small-size effect on attentional processing speed [F(1, 234) = 5.310, *p* = 0.022, *η*^2^*_p_* = 0.022]. Figure 7 shows the effects of both. Meanwhile, their interaction did not have a significant effect on TMT-A [F(1, 234) = 1.760, *p* = 0.186, *η*^2^*_p_* = 0.007]. These findings indicate that transitioning from early to middle adolescence improves attentional processing speed, and that high gaming skill indicates a better attentional processing speed than low gaming skill.

**Figure 8:**
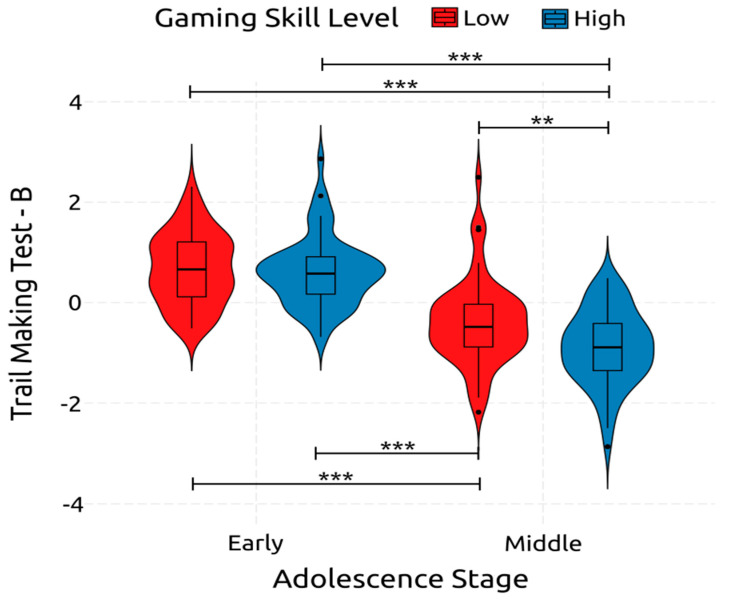
Comparisons in the interaction between adolescence stage and gaming level: mental flexibility; ** *p* ≤ 0.01, *** *p* ≤ 0.001.

Adolescence had a statistically significant and large effect on TMT-B, measuring cognitive flexibility [F(1, 234) = 173.280, *p* < 0.001, *η*^2^*_p_* = 0.425]. It was also significantly affected by gaming skill with a small effect size [F(1, 234) = 8.200, *p* = 0.005, *η*^2^*_p_* = 0.034]. Furthermore, the interaction between gaming skill and adolescence significantly affected cognitive flexibility, with a small effect size [F(1, 234) = 4.85, *p* = 0.029, *η*^2^*_p_* = 0.020]. All these are depicted in Figure 8. These results indicate that growing up from early to middle adolescence increases cognitive flexibility and that improved gaming skill leads to better cognitive flexibility.

Furthermore, post hoc comparisons revealed interesting interactions. early-low compared to early-high was non-significant [*t*(234) = −0.468, *p* = 1, *d* = −0.086]. Early-high compared to both middle-low and middle-high achieved significance, with both achieving a large effect [*t*(234) = 10.820, *p* < 0.001, *d* = 1.992] and [*t*(234) = 7.283, *p* < 0.001, *d* = 1.335], respectively. The same can be observed in the interactions between early-low, and middle-low and middle-high, with both achieving significant and large effects [*t*(234) = 11.333, *p* < 0.001, *d* = −2.078] and [*t*(234) = 7.784, *p* < 0.001, *d* = 1.421], respectively. Finally, comparing middle-low and middle-high, the effect was significant and of medium size [*t*(234) = 3.582, *p* = 0.002, *d* = 0.657]. Considering the post hoc comparisons, when both age and gaming skill are considered simultaneously, it can be pointed out that early adolescents with low gaming skill do not differ in cognitive flexibility from those with high gaming skill. However, early adolescents with low gaming skill have significantly lower cognitive flexibility than middle adolescents with low gaming skill and middle adolescents with high gaming skill. Furthermore, early adolescents with high gaming skill have significantly lower cognitive flexibility than middle adolescents with low gaming skill and middle adolescents with high gaming skill. Finally, middle adolescents with low gaming skill have significantly worse cognitive flexibility than those with high gaming skill.

**Figure 9:**
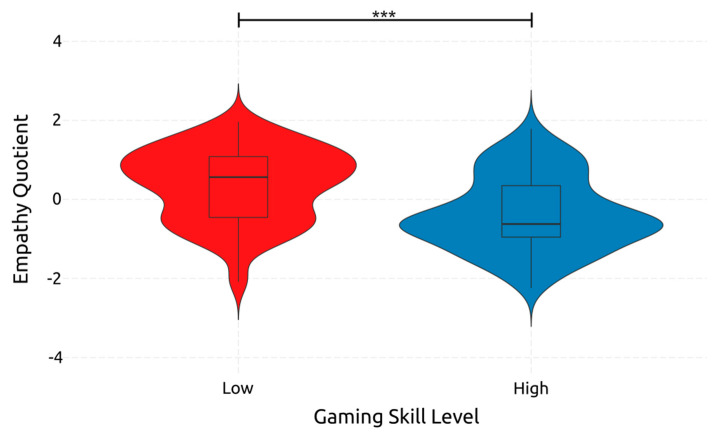
Comparison of low vs. high gaming skill level: empathy quotient; *** *p* ≤ 0.001.

Regarding EQ, and thus empathy, only gaming skill had a significant, medium-sized effect on it, which can also be seen in Figure 9 [F(1, 234) = 37.544, *p* < 0.001, *η*^2^*_p_* = 0.138]. Adolescence had a non-significant effect on empathy [F(1, 234) = 0.003, *p* = 0.954, *η*^2^*_p_* = 0.000]. Also, the interaction between adolescence and gaming skill had a non-significant effect on empathy [F(1, 234) = 0.019, *p* = 0.889, *η*^2^*_p_* = 0.000]. These results indicate that adolescents with higher gaming skill have lower empathy than those with lower gaming skill. At the same time, the adolescence stage does not significantly affect adolescents’ empathy.

**Figure 10:**
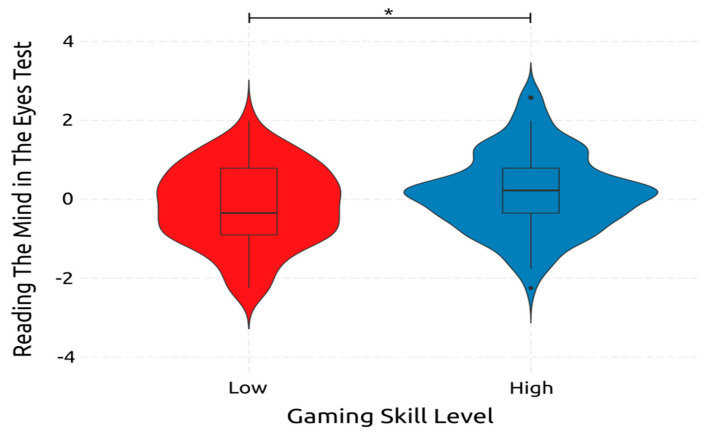
Comparison of low vs. high gaming skill level: emotion recognition; * *p* ≤ 0.05.

Finally, RMET and, subsequently, emotion recognition ability, was only significantly affected by gaming skill with a small effect size [F(1, 234) = 37.544, *p* < 0.001, *η*^2^*_p_* = 0.138]. Adolescence did not significantly affect the emotion recognition ability of adolescents [F(1, 234) = 0.003, *p* = 0.954, *η*^2^*_p_* = 0.000]. Also, adolescence gaming skill interaction did non-significantly affect the score of RMET [F(1, 234) = 0.019, *p* = 0.889, *η*^2^*_p_* = 0.000]. The effect of gaming skill on RMET is demonstrated in Figure 10. These findings indicate that more highly skilled adolescent gamers have better emotion recognition abilities than lower-skilled ones. On the contrary, the age of adolescents does not affect their ability to recognize emotions.

## 4. Discussion

The present study had two aims. The first was the development, implementation, and assessment of GSQ, a tool designed to comprehensively assess adolescents’ videogame skills in the six most common gaming genres. The other aim of this study was to investigate the effect of skills in different gaming genres on seven cognitive skills: language, verbal fluency, executive functions, memory, visuospatial ability, attentional processing speed and cognitive flexibility. Furthermore, the effects of the said gaming skills were also identified on two affective abilities, namely emotion recognition and empathy. High gaming skills were consistently associated with enhanced cognitive abilities and emotion recognition, whereas they correlated with lower levels of empathy. The results indicated that GSQ is a highly reliable and valid tool that can be used to succinctly and accurately assess the gaming skills of an adolescent in the most common gaming genres. At the same time, gaming skills were found to significantly influence memory, executive functions, overall cognition, attentional processing speed, empathy, emotion recognition ability and cognitive flexibility. Especially in the case of cognitive flexibility, it was found that the interaction between gaming skill (low versus high) and stage of adolescence (early versus middle) was significant.

### 4.1. Validatity and Relialibility of the Gaming Skills Questionnaire

The development and validation of the GSQ represent a significant contribution to the field of video game research. GSQ’s comprehensive assessment of gaming skills across the six most common gaming genres fills a crucial gap in the existing literature, providing researchers with a valuable tool to investigate individuals’ gaming skills and frequency of play within these genres. The tool indicated high Cronbach’s α coefficients for each section of GSQ corroborating its strong internal consistency, suggesting that the questionnaire consistently measures the same underlying constructs. This is a fundamental aspect of any reliable measurement tool, emphasizing the utility of GSQ in assessing gaming abilities.

The CFA further supported the robustness of GSQ’s construct validity. GSQ effectively captures the intended dimensions of gaming skills across the six genres. The strong convergent validity, demonstrated by substantial loadings of all items, reinforces GSQ’s effectiveness in measuring gaming skills. Lastly, the excellent divergent validity, as indicated by low associations between GSQ sections, highlights the questionnaire’s ability to discriminate between different aspects of gaming skills. Together, these findings suggest that the questionnaire accurately reflects the constructs it was designed to evaluate, captures distinct dimensions of gaming skills within each genre preventing overlap in the assessment, and provides an accurate and valid assessment of adolescents’ gaming abilities.

### 4.2. Gaming Skills Effects on Cognitive and Affective Functioning in Adolescence

#### 4.2.1. Language

Language proficiency, measured via the ECAS-language sub-score, was examined in relation to adolescents’ age, gaming skills, and parental education level. Results indicated that age significantly influences adolescents’ language ability, with an older age indicative of better language ability. This effect was also supported by the finding that age is a significant predictor of language in the best regression model. The fact that age influences adolescents’ language skill aligns with the existing literature [125,126]. The other predictor included in the best regression model for language was parental education level, whose increase indicated an increase in the language ability of adolescents. This is also consistent with literature, as it has been found that parents of higher education tend to have children with improved language skills [7]. However, gaming ability did not influence adolescents’ language skills. This finding is consistent with some studies that found no association between gaming and language skills of adolescents [65] but contrasts with other studies which indicated that videogame play can be detrimental to an adolescent’s language abilities [66]. Finally, language was also correlated with parents’ age, AGS, PGS, and TGS.

#### 4.2.2. Verbal Fluency

The ECAS-verbal fluency ANOVA revealed a significant difference between the verbal fluency of adolescents in early versus middle adolescence, with the latter group having a superior verbal fluency score. This was also apparent in the regression analysis, as age was a highly contributing predictor in the best regression model. The fact that adolescents’ age correlates positively with verbal fluency is in accordance with the literature [127]. Two more predictors were included in the best verbal fluency regression model: residence location and SpGS. To our knowledge, evidence is absent on the effect of the population size of the place of residence on adolescents’ verbal fluency skills. This novel finding could be investigated in follow-up studies, exploring how the population size of one’s residence affects cognitive skills. Moreover, there is a lack of studies investigating sports games and verbal fluency, thus providing another subject for further investigation. Lastly, it must be stated that verbal fluency was correlated with the age of the participants and their parents’ and with PGS.

#### 4.2.3. Executive Functioning

Findings regarding executive functioning, measured by ECAS-executive functioning, indicated highly skilled gamers exhibited better executive functions than lowly skilled gamers, corroborating existing literature [51,55,56,57]. The best regression model revealed that all three predictors, AGS, StGS, and PGS, positively influenced executive functions. Aligning with previous research, these genres, Action-Adventure, Strategy, and Puzzle games, require cognitive investment from player to enhance their skills, resulting in significant improvements in executive functions [57,58]. Some research has even named strategy games significantly better at training executive functions [57]. It must be noted, though, that action videogames seem to contribute more to improving executive functions than puzzle videogames, according to the best regression model produced, which is not in line with some research [104]. Furthermore, these findings add to the literature disproving the notion that commercially available videogames do not sufficiently train executive functions to induce improvements in them [60]. Finally, it must be noted that the ECAS-executive function score was significantly correlated with the age of the participants, their residence location, their parents’ age, the FPSGS, the RPGS, the AGS, the StGS, the PGS, and the TGS.

#### 4.2.4. Memory

Memory, derived from the ECAS-memory sub-score, featured a significant difference when comparing high to low-skilled gamers and early to middle adolescents. Adolescents with high gaming skills have better memory than those with low gaming skills, which is in accordance with the literature [51,52,53,54,57]. Moreover, adolescents in early adolescence have worse memory scores than those in middle adolescence, a finding supported by the available research [128]. When investigating the best regression model for memory, age, residence location, and TGS were the included predictors. Older age positively influences memory, which, as stated above, is in accordance with the literature [128]. In addition, living in a bigger population center leads to better memory, which disagrees with research that found no difference in adolescents’ memory in rural and urban areas [129]. Finally, ECAS-memory was significantly correlated with participants’ age, residence location, parents’ education, FPSGS, RPGS, AGS, StGS, PGS, and TGS.

#### 4.2.5. Overall Cognition

Analysis of the total cognitive ECAS score revealed significant differences between low and high gaming skill groups and between early and middle adolescence. High-gaming skill individuals exhibited better overall cognition than low skilled ones, consistent with varying effects of different gaming genres on cognitive abilities (language, verbal fluency, executive functioning, memory) discussed earlier. Additionally, research points out that videogame play has a net positive effect on the cognition of adolescents [27,90,130,131]. Teens in middle adolescence also seemed to have better overall cognition scores than those in early adolescence, with research supporting this outcome [31,126,128,132]. Moreover, this is also in accordance with the best regression model, as age was a significant predictor, indicating that higher age led to better ECAS-Total score. Apart from age, the other predictors included in the best regression model of total cognition were residence location and TGS score. Evidence suggests that the place of residence may impact cognition, as indicated in a study that differentiated between various neighborhoods; however, it primarily focused on the economic level of neighborhoods rather than their population size [9]. Further research is required to explore the relationship between residing in larger population centers and its potential impact on overall cognition. Finally, the ECAS-Total Score was correlated significantly with the participants’ age and place of residence, the age and education of the participants’ parents, FPSGS, RPGS, AGS, StGS, PGS, and TGS.

#### 4.2.6. Attentional Processing Speed

Attentional processing speed, as assessed by the TMT-A results, demonstrated that highly skilled gamers had better attentional processing speed than low-skilled gamers, in line with previous research [36,39,130,133,134,135,136]. Additionally, findings revealed that participants in middle adolescence achieved better outcomes in the TMT-A than those in early adolescence. This finding was supported by the fact that the best regression model for TMT-A included only the age of the participants as a predictor, with older adolescents tending to exhibit a better attentional processing speed. Finally, the TMT-A score was significantly correlated with the age of the participants and their parents, AGS, PGS, and TGS.

#### 4.2.7. Cognitive Flexibility

The cognitive flexibility, which was measured by TMT-B, did exhibit interesting outcomes. Early adolescents appeared to have significantly less cognitive flexibility than medium adolescents, and low-skilled gamers seemed to have significantly less cognitive flexibility than highly skilled gamers. In addition, there were significant interactions between the participants’ age and their gaming skills. Middle adolescents with high gaming skills displayed better cognitive flexibility than did middle adolescents with low gaming skills, early adolescents with high gaming skills, and early adolescents with low gaming skills. In turn, middle adolescents with low gaming skills had better cognitive flexibility than early adolescents with high gaming skills and early adolescents with low gaming skills. Finally, there were no significant differences between early adolescents with high and low gaming skills. These results indicate that age and gaming skills are important in shaping an adolescent’s cognitive flexibility and that the effect of gaming is greater than the effect of age. Age and TGS have also been a part of TMT-B’s best regression model, further increasing the weight of these findings.

Age is well-documented in how it influences cognitive abilities, and the present findings are in accordance with the published literature, namely that the older an adolescent, the better their cognitive abilities [137]. Furthermore, gaming, in general, has been documented to improve the cognitive flexibility of the players [46,47,48,138,139]. However, the findings of the present study did not find differences between genres, something that has been indicated by literature, with genres like strategy, FPS, and action-adventure offering the biggest improvements [45,48]. The third and final predictor for TMT-B was residence location, with cognitive flexibility being improved when residing in more populated areas, although there is a lack of studies investigating this. Finally, the TMT-B scores were significantly correlated with the age of the participants and their parents, the family income, the AGS, StGS, PGS, and TGS.

#### 4.2.8. Emotion Recognition

The ANOVA results of RMET, which measured emotion recognition, indicate that highly skilled gamers possess better emotion recognition abilities than low-skilled gamers. Furthermore, four of the five predictors of the best regression model of RMET performance were gaming skills: SpGS, StGS, AGS, and PGS. However, the effect on emotion recognition abilities was negative for the former two, while it was positive for the latter two gaming genres. It can be hypothesized that the contents of the games are significantly different in that they lead to different results regarding emotion recognition, a fact that is already indicated by relevant research [89,90,92,140]. The fifth predictor in the best regression model for RMET was residence location, with living in a more populated area indicative of a better emotion recognition ability. However, there has been no research on this subject, thus providing an interesting subject for future research. Finally, the RMET scores were significantly correlated with the participants’ residence location, SpGS, AGS, PGS, and TGS.

#### 4.2.9. Empathy

An ANOVA on empathy measured by the EQ revealed that participants with high gaming skills exhibited lower levels of empathy than those with low gaming skills. This finding can be partly attributed to the negative effect of violent videogames on empathy [77,79,141]. However, this is in contrast to a body of research that indicates that some videogames, either those that are prosocial in nature like RPGs [72,80,81] or those that require players to cooperate [142], tend to cause increased empathy in their players [143]. The ANOVA results were also reinforced by the best regression model produced, with one of the two predictors included being TGS. The other predictor is residence location, which seems to be indicative of empathy, with rural, less populated area residents having more empathy than those in urban, more populated ones [8]. Finally, empathy was correlated significantly with the age and location of residence of the participants, and all the gaming skills, including SpGS, FPSGS, RPGS, AGS, StGS, PGS, and TGS.

### 4.3. Limitations, Implications, and Future Studies

The present study has some limitations. First, gaming, in general, has become a very broad medium, and thus, categorizing each game in a genre can be difficult, with games belonging to a particular genre featuring significant differences. This study included six of the most common gaming genres to cover most players, but a more extensive gaming categorization could have merits. For example, distinguishing FPS games into more casual games and those more akin to military simulation could have produced different results. Additionally, while the present study strived to include many cognitive and affective abilities, far more could be investigated in future research. Furthermore, with the development of the GSQ, future studies should investigate adolescents’ different abilities, or target a diverse audience, like young adults or even younger children. Finally, considering the wide use of video games even in adulthood, while the GSQ used was found to be a valid and reliable tool for assessing self-reported gaming skills in adolescents, future studies should strive to examine GSQ’s psychometric properties in adults.

Building upon these limitations and considering the potential for future research, it is essential to recognize the significance of the present study’s findings and their implications for understanding the complex interactions between video games and cognitive and affective abilities. The absence of other tools, to the best of our knowledge, designed to comprehensively and succinctly measure an adolescent’s gaming skills, including across various game genres, underscores the importance of developing and validating the GSQ for future studies in the area. Furthermore, the results of the research highlight the nuanced effects of video games on adolescents’ cognitive and affective abilities, with each gaming genre exerting unique influences on different aspects of cognition, with most effects being positive. These findings contribute to the growing body of evidence suggesting that video games can have positive effects on cognitive functioning, challenging previous negative perceptions and emphasizing their potential benefits for various cognitive domains.

## 5. Conclusions

The aim of this study was twofold. First, it created a tool that can be used to collect data regarding the videogame skills of a person in the six most common genres. GSQ is a highly reliable and valid tool that can be used in a plethora of videogame research projects. The creation of a succinct and robust way to quantify the gaming skills of an individual in the most common gaming genres is expected to contribute to the ever-increasing literature on the extremely popular topic of videogames. Additionally, the other goal of the present study was to uncover the different ways videogame play impacts cognitive and affective abilities. Some cognitive functions, such as language and verbal fluency, were not affected by the different levels of gaming skills of adolescents. Other cognitive and affective abilities improved in adolescents with high gaming skills. These included memory, executive functions, attentional processing speed, total cognition, and emotion recognition ability. Empathy, on the other hand, was the only ability tested which was diminished in highly skilled videogame players. In the case of cognitive flexibility, the interaction between age and gaming skill uncovered that, while both older age and a higher gaming skill indicate improvement in cognitive flexibility, the role of gaming is greater than that of age. Finally, specific video game genres uniquely impact particular cognitive abilities. Verbal fluency is negatively affected by sports game skills, while executive function benefits from proficiency in action-adventure, strategy, and puzzle games. Additionally, emotion recognition is positively associated with skills in action-adventure and puzzle games but negatively linked to sports and strategy game skills.

## Figures and Tables

**Table 1:** Descriptive statistics: demographics, gaming skills, cognitive and affective functioning.

	Mean	SD	Minimum	Maximum
Age	13.63	1.43	12	16
Residence Location (Median/Mode)	2/2	-	1	4
Parent Age	43.89	4.49	36	55
Parent Education	15.31	1.78	12	18
Family Income (Median/Mode)	3/3	-	3	4
Sport Games Skill	3.13	1.69	2	8
FPS Games Skill	2.99	1.55	2	9
RPG Skill	2.71	1.46	2	11
Action-Adventure Games Skill	3.35	1.85	2	10
Strategy Games Skill	2.48	1.49	2	10
Puzzle Games Skill	3.38	1.72	2	9
Total Gaming Skill	18.05	4.52	12	33
ECAS–Language	25.54	1.15	22	28
ECAS–Verbal Fluency	19.11	4.38	6	24
ECAS–Executive Functions	40.89	2.25	34	47
ECAS–Memory	19.19	2.47	13	24
ECAS–Visuospatial	12.00	0.00	12	12
ECAS–Total Score	116.73	7.52	89	135
TMT–A–Reaction Time	36.34	6.85	22.70	49.30
TMT–B–Reaction Time	96.90	40.78	30.70	180.50
RMET	20.59	1.81	16	25
Empathy Quotient	41.46	6.00	28	53

FPS = first-person shooting; RPG = role-playing games; ECAS = Edinburgh Cognitive and Behavioural ALS Screen; TMT = Trail Making Test; RMET = Reading the Mind in the Eyes Test.

**Table 2:** Internal reliability and goodness of fit for the GSQ.

Statistic	Threshold	Outcome
Cronbach’s α	≥0.70	SpGS = 0.80
		FPSGS = 0.86
		RPGS = 0.91
		AGS = 0.82
		StGS = 0.83
		PGS = 0.81
χ^2^/df	≤2.00	1.97
CFI	≥0.90	0.959
TLI	≥0.90	0.906
SRMR	≤0.08	0.079
RMSEA	≤0.08	0.078

SpGS = sport games skill; FPSGS = first-person shooting games skill; RPGS = role-playing games skill; AGS = action-adventure games skill; StGS = strategy games skill; PGS = puzzle games skill; CFI = Comparative Fit Index; TLI = Tucker–Lewis Index; SRMR = Standardized Root Mean Residual; RMSEA = Root-Mean-Square Error of Approximation.

**Table 3:** Pearson’s r correlations: cognitive skills, empathy, demographics, and gaming skills.

	ECAS-L	ECAS-VF	ECAS-EF	ECAS-M	ECAS-TS	TMT-A	TMT-B	RMET	EQ
Age	0.69 ***	0.71 ***	0.18 ***	0.23 ***	0.68 ***	−0.79 ***	−0.76 ***	−0.01	−0.14 *
Residence Location ^	−0.09	0.05	0.15 **	0.26 ***	0.14 **	0.02	0.09	0.12 *	−0.27 ***
Parent Age	0.18 ***	0.15 **	0.12 *	0.08	0.23 ***	−0.25 ***	−0.20 ***	0.04	−0.07
Parent Education ^	0.19 ***	0.07	0.09	0.12 *	0.14 *	−0.06	−0.10	0.01	−0.03
Family Income	0.08	0.03	0.01	−0.01	0.05	−0.10	−0.17 **	0.01	−0.07
Sport Games Skill	0.07	−0.07	−0.05	0.07	0.01	−0.06	−0.04	−0.12 *	−0.13 *
FPS Games Skill	0.05	0.05	0.12 *	0.18 *	0.11 *	−0.06	−0.05	0.07	−0.21 ***
RPG Skill	0.07	0.02	0.23 ***	0.19 ***	0.17 **	0.03	−0.08	0.08	−0.19 ***
Action-Adventure Games Skill	0.11 *	0.08	0.33 ***	0.21 ***	0.25 ***	−0.13 *	−0.15 **	0.21 ***	−0.37 ***
Strategy Games Skill	0.09	0.07	0.17 **	0.16 **	0.17 **	−0.09	−0.11 *	−0.08	−0.14 **
Puzzle Games Skill	0.18 ***	0.16 ***	0.35 ***	0.23 ***	0.33 ***	−0.18 ***	−0.28 ***	0.17 **	−0.26 ***
Total Gaming Skill	0.19 ***	0.10	0.38 ***	0.35 ***	0.35 ***	−0.19 ***	−0.24 ***	0.11 *	−0.48 ***

ECAS = Edinburgh Cognitive and Behavioural ALS Screen; L = language; VF = verbal fluency; EF = executive functions; M = memory; TS = total score; TMT = Trail Making Test; RMET—Reading the Mind in the Eyes Test; EQ = empathy quotient; FPS = first-person shooting; RPG = role-playing games; * *p* ≤ 0.05, ** *p* ≤ 0.01, *** *p* ≤ 0.001; ^ Spearman’s Rho values in this row.

**Table 4:** Best regression models for predicting cognitive and affective abilities.

Predicted	Predictors	β Coefficient	*p*-Value (β)	R^2^
ECAS–Language	Age	0.68	<0.001 ***	0.50
	Parent Education	0.13	<0.001 ***	
ECAS–Verbal Fluency	Age	0.72	<0.001 ***	0.52
	Residence Location	0.08	0.026 *	
	Sport Games Skills	−0.11	0.004 **	
ECAS–Executive Functions	Action Games Skill	0.29	<0.001 ***	
	Strategy Games Skill	0.16	0.002 **	0.21
	Puzzle Games Skill	0.24	<0.001 ***	
ECAS–Memory	Age	0.19	<0.001 ***	0.20
	Residence Location	0.23	<0.001 ***	
	Total Gaming Skill	0.28	<0.001 ***	
ECAS–Total Score	Age	0.66	<0.001 ***	0.54
	Residence Location	0.15	<0.001 ***	
	Total Gaming Skill	0.21	<0.001 ***	
Trail Making Test–A	Age	−0.79	<0.001 ***	0.63
Trail Making Test–B	Age	−0.74	<0.001 ***	0.60
	Family Income	−0.10	0.003 **	
	Total Gaming Skill	−0.11	0.002 **	
Reading the Mind in the Eyes Test	Residence Location	0.11	0.046 *	0.10
	Sport Games Skills	−0.14	0.007 **	
	Action-Adventure Games Skill	0.14	0.014 *	
	Strategy Games Skill	−0.13	0.021 *	
	Puzzle Games Skill	0.17	0.003 **	
Empathy Quotient	Total Gaming Skill	−0.45	<0.001 ***	0.27
	Residence Location	−0.21	<0.001 ***	

ECAS = Edinburgh Cognitive and Behavioural ALS Screen; * *p* ≤ 0.05, ** *p* ≤ 0.01, *** *p* ≤ 0.001.

**Table 5:** Cognitive and affective functioning per adolescence stage and gaming skill level.

	Adolescence Stage	Gaming Skill	Mean	SD	Minimum	Maximum
Age	Early	High	12.4	0.50	12	13
		Low	12.3	0.48	12	13
	Middle	High	15.1	0.84	14	16
		Low	14.8	0.78	14	16
Total Gaming Skill	Early	High	21.1	3.93	17	32
		Low	14.5	1.48	12	16
	Middle	High	21.6	4.15	17	33
		Low	14.5	1.54	12	16
ECAS–Language	Early	High	24.6	0.67	23	26
		Low	24.7	0.82	23	26
	Middle	High	26.3	0.98	23	28
		Low	26.1	0.88	24	27
ECAS–Verbal Fluency	Early	High	15.2	4.57	6	22
		Low	16.1	3.86	8	20
	Middle	High	21.5	1.91	16	24
		Low	21.8	1.88	18	24
ECAS–Executive Functions	Early	High	41.2	2.03	34	44
		Low	40.1	2.29	35	45
	Middle	High	41.7	2.42	37	47
		Low	40.3	1.67	36	45
ECAS–Memory	Early	High	19.2	2.41	14	24
		Low	18.6	2.11	14	23
	Middle	High	20.3	2.27	15	24
		Low	19.0	2.30	14	23
ECAS–Visuospatial	Early	High	12.0	0.00	12	12
		Low	12.0	0.00	12	12
	Middle	High	12.0	0.00	12	12
		Low	12.0	0.00	12	12
ECAS–Total Score	Early	High	112.2	7.85	89	125
		Low	111.6	5.98	97	122
	Middle	High	121.8	5.19	111	135
		Low	119.2	3.78	110	129
TMT–A–Reaction Time	Early	High	42.0	4.93	24.5	49.30
		Low	42.5	3.90	31.8	48.00
	Middle	High	31.0	4.88	22.7	46.40
		Low	33.3	4.63	24.0	43.70
TMT–B–Reaction Time	Early	High	129.2	25.05	66.80	180.50
		Low	131.9	27.11	76.10	172.90
	Middle	High	66.5	28.46	30.70	130.70
		Low	84.4	34.52	31.40	176.00
RMET	Early	High	21.0	1.51	18	25
		Low	20.1	1.90	16	23
	Middle	High	20.7	2.00	16	25
		Low	20.5	1.83	16	24
Empathy Quotient	Early	High	39.5	5.09	32	51
		Low	43.8	5.34	34	53
	Middle	High	39.3	6.03	28	52
		Low	43.8	5.60	29	52

ECAS = Edinburgh Cognitive and Behavioural ALS Screen; TMT = Trail Making Test; RMET = Reading the Mind in the Eyes Test.

## Data Availability

The data presented in this study are available on request from the corresponding author. The data are not publicly available due to the ethical approval requirements.

## Supplementary Materials

The GSQ (English version) can be accessed here: http://dx.doi.org/10.13140/RG.2.2.27257.24160 accessed on 10 January 2024.
